# Psychological and Emotional Recognition of Preschool Children Using Artificial Neural Network

**DOI:** 10.3389/fpsyg.2021.762396

**Published:** 2022-02-08

**Authors:** Zhangxue Rao, Jihui Wu, Fengrui Zhang, Zhouyu Tian

**Affiliations:** ^1^School of Education, China West Normal University, Nanchong, China; ^2^College of Life Science, Sichuan Agricultural University, Yaan, China; ^3^School of Economics and Management, Shenyang Institute of Technology, Fushun, China

**Keywords:** traditional education, artificial neural network, emotion recognition, preschool children, education

## Abstract

The artificial neural network (ANN) is employed to study children’s psychological emotion recognition to fully reflect the psychological status of preschool children and promote the healthy growth of preschool children. Specifically, the ANN model is used to construct the human physiological signal measurement platform and emotion recognition platform to measure the human physiological signals in different psychological and emotional states. Finally, the parameter values are analyzed on the emotion recognition platform to identify the children’s psychological and emotional states accurately. The experimental results demonstrate that the recognition ability of children aged 4–6 to recognize the three basic emotions of happiness, calm, and fear increases with age. Besides, there are significant age differences in children’s recognition of happiness, calm, and fear. In addition, the effect of 4-year-old children on the theory of mind tasks is less than that of 5- to 6-year-old children, which may be related to more complex cognitive processes. Preschool children are experiencing a stage of rapid emotional development. If children cannot be guided to reasonably identify and deal with emotions at this stage, their education level and social ability development will be significantly affected. Therefore, this study has significant reference value for preschool children’s emotional recognition and guidance and can promote children’s emotional processing and mental health.

## Introduction

The preschooler stage is a critical period for the fast development of the emotion recognition ability of children. Attractive interaction and continuous and active peer interaction are primary development tasks of education in the preschooler stage, and emotion recognition ability is the paramount development task. The development of social acceptability of children depends on the development of recognition and understanding of other people’s emotions in the preschooler stage. Lu et al. (2019) believed that children’s emotion management refers to a skill that could transform or serve children’s social ability from emotion recognition, control, and improvement to the final purpose. They provided scientific suggestions for the cultivation and improvement of children’s emotion management ability through the investigation of different types of kindergartens (Lu et al., 2019). Bao (2021) mentioned in his research that the causes of children’s emotional problems were diverse, and children had many types of emotional symptoms and lack of typicality. On this basis, the author focused on the early identification of children’s emotional problems and corresponding countermeasures from the perspective of the importance and necessity of early identification of children’s emotional problems (Bao, 2021). Rapid and accurate recognition of other people’s emotions and positive response in social life are conducive to the better survival and development of individuals. In the preschool period, lacking the ability to accurately express or recognize emotions will seriously affect children’s academic growth and interpersonal relationship. Therefore, it is vitally important to study the development of the emotion recognition ability of preschool children. In emotion recognition, explicit facial expression information is the primary source of clues for individuals.

There are generally two emotion recognition methods categories: physiological signal recognition and non-physiological signal recognition. When people produce certain emotions or changes, their nervous systems will stimulate some organs and muscles of the human body to produce related physiological activities and change some physiological signals of the human body. The physiological signal recognition method of emotion indirectly reflects the corresponding emotional state by measuring the physiological signals of the human body, such as electro-cardio signal (ECG), electroencephalogram (EEG), galvanic skin response (GSR), and respiration (RSP). Multitudes of experiments and studies have confirmed the scientificity of emotion recognition using physiological signals. Scholars in related fields have successfully identified eight different emotion types, such as happiness, anger, sadness, and fear, by collecting and analyzing the changes of a series of physiological signals. Compared with the non-physiological signal recognition method, the physiological signal recognition method has better accuracy and objectivity, with no restrictions on the applicable population. Therefore, the emotion recognition method based on human physiological signals has high research and practical value.

In the present work, the psychological emotion recognition of preschool children based on artificial neural network (ANN) under the background of traditional education is deeply discussed, combined with the actual situation of preschool children. The emotion recognition platform based on the backpropagation neural network (BPNN) model is used to study the emotion recognition status of preschool children. Then, the characteristic parameters of physiological signals under different emotions of children aged 4–6 are analyzed and investigated. Finally, emotion recognition research is carried out on the test samples. The research content can provide theoretical and practical guidance for improving preschool children’s emotional recognition ability and mental health for better development. In the traditional education system, preschool children’s education is highly critical. Optimizing the children’s mental health education system can improve children’s comprehensive quality and meet the growing needs of modern children. In the practice of preschool education, it is essential to actively explore their psychological emotions, seek an effective route of mental health education, and guide children’s healthy growth. In developing children’s mental health education, it is necessary to select educational methods and paths by combining modern information equipment and the characteristics of mental health education. Besides, mental health education activities of young children are the top priority of preschool education. Attention to children’s mental health is also crucial in school health education.

Previous studies on preschool children’s psychological emotion recognition mainly focus on the external aspects such as the environment and teaching, achieving specific outcomes. However, there are few studies on the application of ANN to emotion recognition of preschool children in China. Therefore, the ANN-based emotion recognition is discussed here to provide strong support for the related research field, enrich the relevant research content, and afford reference for subsequent research.

## Literature Survey

At present, there have been many studies on psychological emotion recognition through human electrophysiological signals worldwide. The physiological signals used for research mainly include electrocardiography (ECG), EEG, GSR, RSP, blood volume pulse (BVP), electromyography (EMG), and skin temperature signal (STS). Pan et al. (2020) proposed an emotion recognition method based on multiple physiological signal fusion and FCA-Relief feature selection to solve the problems of many feature dimensions and low recognition rate in the current emotion recognition research. They fused the physiological signal features extracted from the two dimensions of the time domain and frequency domain as the classifier’s input for emotion classification (Pan et al., 2020). Chen et al. (2020) put forward an emotion recognition model to extract the features of physiological signals such as photoplethysmography, GSR, respiration amplitude, and STS. They also performed the Recursive Feature Elimination-Correlation Bias Reduction-Support Vector Machine to select features and support vector machines for classification (Chen et al., 2020). In addition, Donghua University and Jiangsu University have studied the emotional recognition of physiological signals similar to most studies, such as GSR, ECG, RSP, and SKS; they usually adopted traditional experimental statistical methods and achieved a final recognition rate of more than 70%, obtaining good practical results.

From the above research results, analyzing multiple physiological signals of the human body can more precisely recognize the emotional state. On the other hand, for the emotional recognition of physiological signals, under the same conditions, the more characteristic parameters extracted from physiological signals for emotional recognition, the better the emotional recognition effect. On the other hand, different research employs various algorithms to establish the emotion recognition model. Support Vector Machine algorithm, K-nearest neighbor algorithm, and wavelet analysis algorithm are commonly used. In the aspect of human electrophysiological signal acquisition, there are very mature and perfect acquisition equipment, which can help researchers accurately measure human physiological signals. New research on human physiological signal acquisition equipment is also emerging.

## Materials and Methods

### Emotion Comprehension

Emotion is a significant component of individual psychological experience. Emotion has the function of establishing, maintaining, and changing relationships between individuals and the outside world. This emotion function is considered a kind of ability, namely emotional ability. Emotional comprehension in emotional capacity is an integral part of psychological theory. It refers to emotion processing (e.g., emotional state and regulation), conscious understanding, or understanding how emotion works. The research on emotional comprehension of children in the psychological theory mainly includes children’s recognition of simple emotional expressions and the situations that cause these emotions, understanding of the relationship between emotion and desire, knowing that desire is the cause of emotions, knowledge of the relationship between belief and emotion, knowing belief is the cause of emotions, and understanding of conflict emotion.

Therefore, the research on emotion comprehension of children in the psychological theory focuses on the development of emotion comprehension at different levels. These levels cover expression recognition, that is, children’s recognition of simple expressions and situations causing these emotions; desire emotion comprehension, namely understanding the relationship between emotion and desire, and knowing that desire is the leading cause of emotion; belief emotion comprehension, i.e., understanding the relationship between belief and emotion, and knowing that belief is the cause of emotions; understanding of conflict emotions. There are few studies on children’s emotion comprehension, and existing research methods are relatively single. Previous studies mainly adopt experimental approaches using pictures as materials and supplemented by corresponding objects to identify children’s emotional reactions.

Emotional development is vital to the social development of children. Parent–child communication and peer communication are children’s principal social situations during emotional development. Therefore, developing emotion comprehension is principally affected by parent–child and peer relationships. In short, parents, as supporters and guides in children’s emotional development, their influence changes with children’s age. As equal partners and negotiators, peers gradually form more mature emotion regulation strategies and form groups with specific emotional rules. These specific emotional rules have a unique binding force on children in the group and can change through consultation. Of course, with children’s growth and the influence of parents and peers, children’s emotional development will also be affected by various factors such as social environment, school atmosphere, and mass media.

### Basic Emotion Classification Theory

Primary emotions are generally innate, and different people usually show similar psychological, physiological, and behavioral characteristics under the same basic emotions. Many scholars have put forward their theories on classifying basic emotions. Table 1 lists several famous sorts.

**TABLE 1:** Emotion classification theories.

Scholar	Emotion classification
William James	Anger, fear, sadness, love, etc.
Fridja	Happiness, interest, doubt, sadness, desire, surprise, etc.
Izard	Fear, interest, anger, happiness, contempt, disgust, guilt, shame, grief, surprise, etc.
Ekman	Anger, surprise, fear, happiness, sadness, disgust, etc.
Tomkins	Fear, happiness, surprise, contempt, disgust, anger, grief, interest, shame, etc.
Weiner and Graham	Happiness, sadness, etc.
Oatley	Happiness, sadness, anger, disgust, anxiety, etc.

### Artificial Neural Network

**FIGURE 1:**
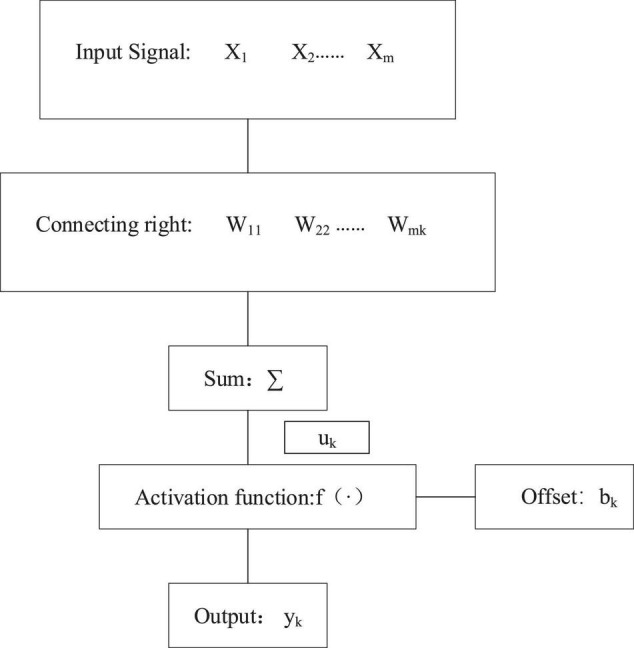
Artificial neuron model.

Artificial neural network is a digital simulation of biological neural networks. Like the nervous system of organisms, ANN is composed of numerous neurons that are basic functional units (Bahareh et al., 2018). Artificial neurons are the basic information processing unit of ANN and the foundation of ANN. Figure 1 illustrates the structure of the artificial neuron model.

The artificial neuron *k* can be expressed as Equations 1, 2.


(1) $$u_{k}=\sum_{i=1}^{m}w_{ik}x_{i}$$

(2) $$y_{k}=f\left(u_{k}+b_{k}\right)$$

Among Equations 1, 2, *w*_*ik*_ represents the link weight between the *k*th neuron and the *i*-th input signal; *u*_*k*_ stands for the weighted sum of the neuronal adder; *b*_*k*_ denotes the bias; *f* (•) refers to the transmission function.

**FIGURE 2:**
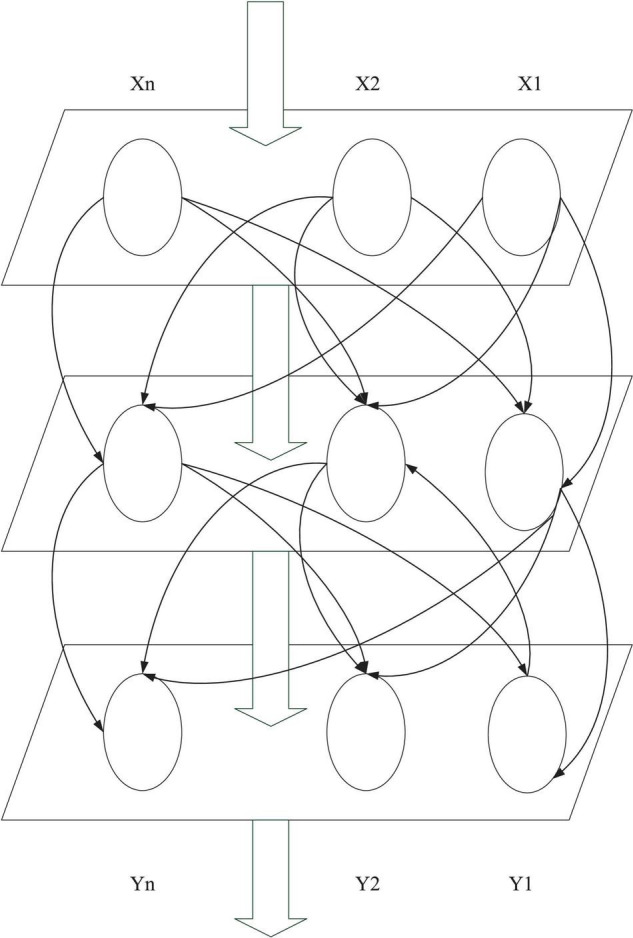
Structure of the simple feedforward hierarchical ANN.

Artificial neural network has various connecting formats and topological structures. Usually, it can be divided into the hierarchical ANN and interconnected ANN. Hierarchical ANNs generally consist of the input, intermediate, and output layers, and each layer is connected sequentially. In addition, the intermediate layer does not directly participate in external input and output, so it is also called the hidden layer. The input layer is connected to the external equipment and sends the input data to the hidden layer. Figure 2 reveals the simple feedforward hierarchical ANN (Chen, 2019; Deepak et al., 2019).

Any two neurons are connected through a particular path in the interconnected ANN. Regardless of the structure of ANN, a single artificial neuron cannot realize information processing. Hence, it is essential to connect substantial artificial neurons according to a particular topological structure and assign the connection weights of each neuron according to specific rules. In this way, a neural network with an information processing function can be constituted (Haytham et al., 2017; Batbaatar et al., 2019).

**FIGURE 3:**
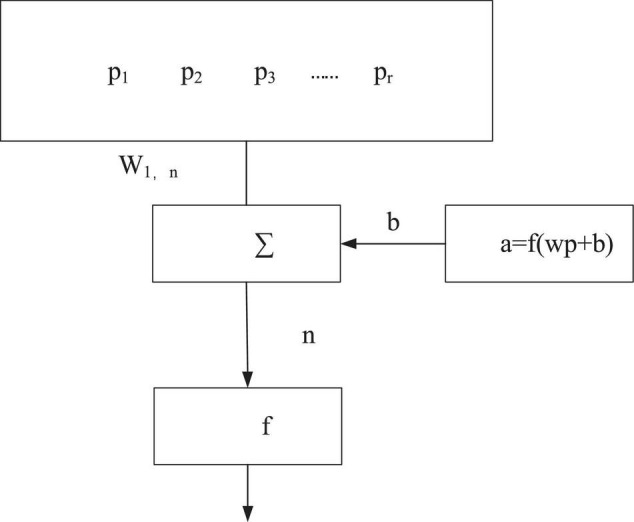
Neuron model of the BPNN.

Backpropagation neural network is a multi-layer feedforward neural network proposed by American psychologists David Everett Rumelhart and James McClelland in 1985. Figure 3 signifies a neuron model of the BPNN (Chen et al., 2018; Shahin et al., 2019).

The neuron model of the BPNN can be written as Equation 3.


(3) a=f(wp+b)

In Equation 3, *a* is the output of BPNN, *p* represents the input of BPNN, and there are *R* input signals. Each input signal has a connection weight *w* connected to the adder. Besides, *f* (•) represents the transmission function of the neuron, which determines the output range of the neuron. Many BP neurons constitute the BPNN (Pawe et al., 2017; Neha et al., 2018).

**FIGURE 4:**
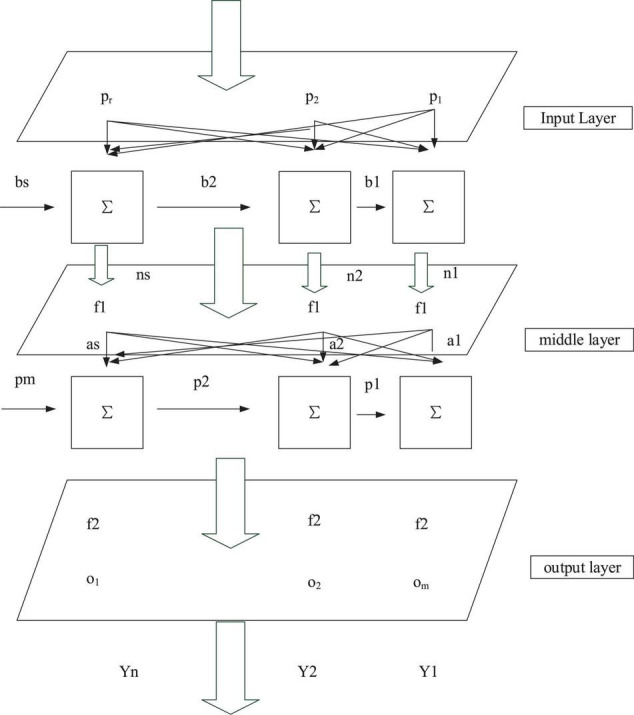
The typical structure of the BPNN model.

Backpropagation neural network is generally composed of an input layer, one or more intermediate layers, and an output layer. Figure 4 indicates the typical structure of a BPNN model.

In Figure 4, the BPNN model has an input layer, an intermediate layer, and an output layer. There are R input signals denoted as p, s neurons in the middle layer, m output signals marked as o. Meanwhile, f1 means the transfer function of the intermediate layer, and f2 refers to the transfer function of the output layer.

**FIGURE 5:**
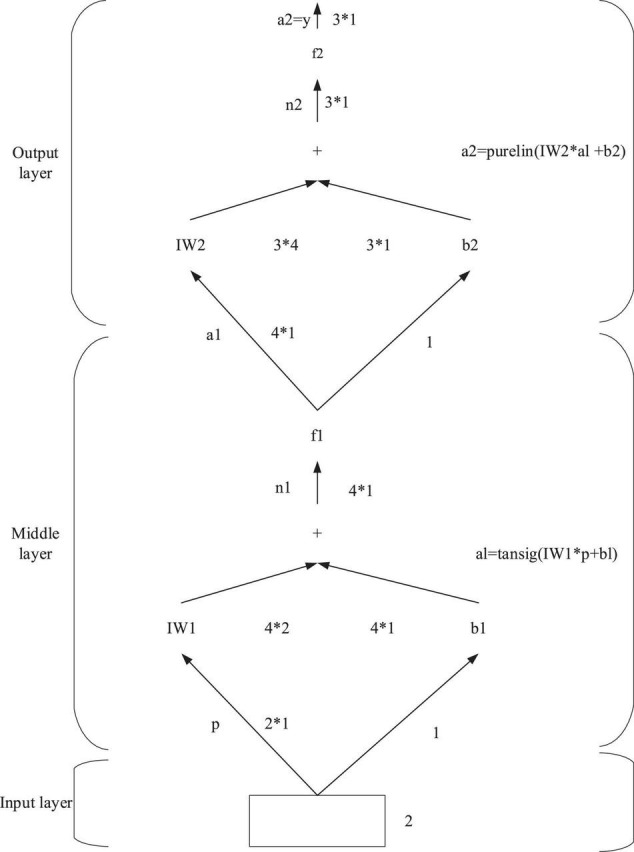
Double-layer BPNN.

The steepest descent backpropagation (SDBP) algorithm based on BPNN is used here to measure the accuracy, and the following example is taken as an illustration. Figure 5 reveals a double-layer BPNN, with two nodes in the input layer, four nodes in the middle layer, and three nodes in the output layer. The transfer function of the intermediate layer is the Sigmoid function named Tansig, and the transfer function of the output layer is the purely linear function.

For such a typical BPNN, assuming that *k* is the number of iterations of training samples of BPNN in the SDBP algorithm, the adjustment and error correction of weights and thresholds in the middle and output layers of BPNN shall be carried out according to Equation 4.


(4) x(k+1)=x(k)-αg(k)

In Equation 4, *x*(*k*) represents the weight vector or threshold vector of each middle layer or output layer of BPNN during the *k*-th training of samples, and *g*(*k*) is determined by Equation 5.


(5) $$g\left(k\right)=\frac{\partial E\left(k\right)}{\partial x\left(k\right)}$$

In Equation 5, *g*(*k*) refers to the gradient vector of the final error of BPNN to the weight vector or threshold vector of each middle and output layer during the *k*-th training of samples, and −*g*(*k*) denotes the opposite direction of the gradient vector, that is, the falling direction of the error. Besides, α represents the learning rate of BPNN, reflecting the speed of BPNN learning samples, *E*(*k*) signifies the final error function of BPNN during the *k*-th training of samples. In general, the last error function is the mean square error. For example, when the number of samples input by BPNN is 1, *E*(*k*) can be expressed as:


(6) $$E\left(k\right)=E\left[e^{2}\left(k\right)\right]\approx \frac{1}{s^{2}}\sum_{i=1}^{s^{2}}{\left[t_{i}^{2}-a_{i}^{2}\left(k\right)\right]}^{2}$$

where:


(7) $$a_{i}^{2}\left(k\right)=f^{2}\left\{\sum_{j=1}^{s^{2}}\left[w_{i,j}^{2}\left(k\right)a_{i}^{1}\left(k\right)-b_{i}^{2}\left(k\right)\right]\right\}$$

that is:


(8) $$a_{i}^{2}\left(k\right)=f^{2}\left\{\sum_{j=1}^{s^{2}}\left[w_{i,j}^{2}\left(k\right)f^{1}\left(\sum_{j=1}^{s^{1}}\left(iw_{i,j}^{1}\left(k\right)p_{i}+ib_{i}^{1}\left(k\right)\right)\right)\right]\right\}$$

then, when there are n samples input into BPNN, there is:


(9) $$E\left(k\right)=E\left[e^{2}\left(k\right)\right]\approx \frac{1}{ns^{2}}\sum_{j=1}^{s^{1}}\sum_{i=1}^{s^{2}}{\left[t_{i}^{2}-a_{i}^{2}\left(k\right)\right]}^{2}$$

According to Equations 4–6, the gradient vector *g*(*k*) of the final error of BPNN to the weight vector or threshold vector of each middle layer and output layer during the *k*-th training is $\frac{\partial E\left(k\right)}{\partial x\left(k\right)}$. Then, *g*(*k*) is substituted into (*x*(*k* + 1) = *x*(*k*)−α*g*(*k*)), the weight and threshold of BPNN can be corrected again, and the output error converges in the decreasing direction. By analogy, continuous iteration can produce a BPNN that meets the requirements. The above method is the SDBP algorithm. The difference between this algorithm and the basic BPNN algorithm is that SDBP adopts the batch processing method, and its modification of the weight threshold of the neuron in the output layer of the intermediate layer is after the sample input and the output error of the neural network, which significantly accelerates the convergence.

### Emotion Recognition Platform Designed Based on Backpropagation Neural Network

**FIGURE 6:**
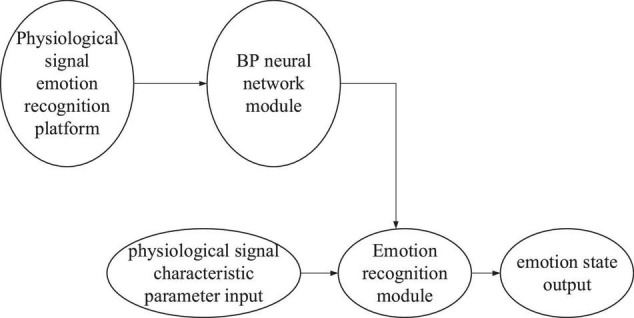
Overall scheme of the emotion recognition platform.

The emotion recognition platform takes the BPNN model as the core, analyzing and calculating the input physiological signals and recognizing emotional states. The inputs into the emotion recognition platform are $\overset{\_\_\_\_}{PR}$ (mean pulse rate), PR-sd (standard deviation of pulse rate), $\overset{\_\_\_\_}{PA}$ (mean pulse amplitude), PA-sd (standard deviation of pulse amplitude), $\overset{\_\_\_\_}{SC}$ (mean skin conductance), SC-sd (standard deviation of skin conductance), $\overset{\_\_\_\_}{RR}$ (mean respiratory rate), RR-sd (standard deviation of respiratory rate), $\overset{\_\_\_\_}{RA}$ (mean respiratory rate), RA-sd (standard deviation of respiratory rate), $\overset{\_\_\_\_\_\_\_}{SKT}$ (mean skin temperature), and SKT-sd (standard deviation of skin temperature). The outputs of the emotion recognition platform involve three emotional states, namely calmness, happiness, and fear. The overall scheme of the platform is presented in Figure 6.

The physiological signal emotion recognition platform comprises the BPNN module, emotion recognition module, and data input/output module (Qian et al., 2018; Jiang et al., 2019). Establishing a suitable BPNN model before using the emotion recognition platform is necessary for physiological signal emotion recognition. A trained BPNN model can calculate the characteristic parameters of physiological signal inputs into the emotion recognition platform and finally output the emotion state result (Raviraj and Ashwinikumar, 2018; Rogoza et al., 2018).

**FIGURE 7:**
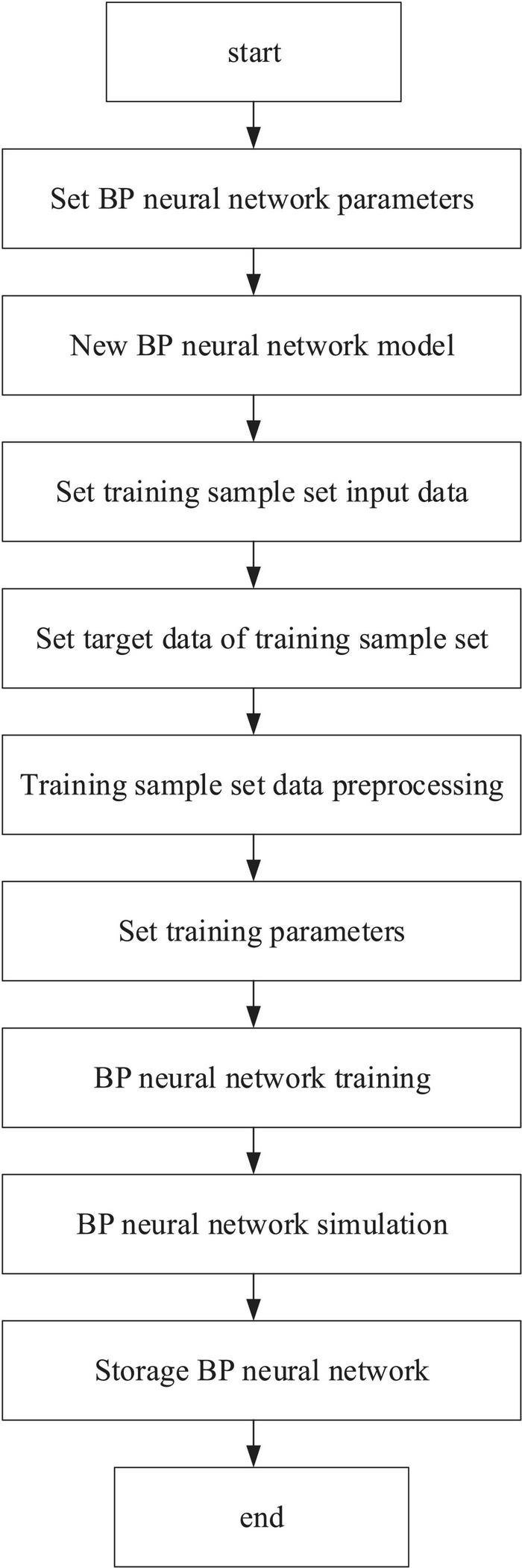
Establishment process of the BPNN model.

In Figure 7, BPNN is the core of the emotion recognition platform to realize the emotion recognition function. Therefore, a suitable BPNN should be established before implementing emotion recognition. The model parameters of the BPNN used for the emotion recognition platform are as follows. There are 12 neurons in the input layer, 1 middle layer in the network, 14 in the middle layer, and 3 in the output layer. Besides, tansig is selected as the transfer function of the intermediate layer, logsig as the transfer function of the output layer, and traingd as the training function. Moreover, the input signal range in the input layer is [0, 1], and the weight learning algorithm and performance function use default values “learngdm” and “mse,” respectively. The BPNN model is established based on the above parameters.

### Experimental Materials and Equipment

Referencing the International Affective Picture System (IAPS) and the domestic revised Chinese Affective Picture System (CAPS), this experiment collects 30 pictures of different contents, such as scenery, animals, food, characters, tools, disability, disaster, and violence, by different ways to induce calmness, happiness, and fear (Zhang et al., 2017; Wu W. et al., 2020). The investigation of emotion recognition reported here is only qualitative research without quantitative research. Therefore, the three-dimensional scoring criteria of IAPS or CAPS are not utilized when selecting the experimental pictures chosen according to the actual effect of emotion elicitation independently. The pictures come from multiple ways, partly from standard emotion recognition systems including CAPS and IAPS, and partly from other emotion-induced pictures used in emotion-induced research. Then, 10 subjects who did not participate in the final experiment are invited to analyze and test the meaning of these 30 pictures and the emotion elicitation. Finally, 15 pictures with sound effects and clear definition are selected as emotion-induced materials in this experiment, and every five emotion-induced pictures correspond to an emotional state (Wu and Wu, 2017; Wu and Song, 2019).

The experimental equipment used in this experiment includes an emotion recognition system (including software and hardware) based on human physiological signals independently designed here and a desktop computer equipped with a 23-inch display of resolution 1920 xten80 for displaying emotion-induced picture materials. The experimental environment is a soundproof room.

### Experimental Subjects and Procedures

Twenty-two children aged 4–6 were selected for this experiment. There are five 4-year-old, eight 5-year-old children, and nine 6-year-old children. Some children quit during the investigation. Consequently, invalid data of a 4-year-old child and a 5-year-old child is excluded, and there remains valid data of 20 children. All children participate voluntarily, with fluent Mandarin, normal movement development, mental health, and regular communication ability with others. After the experiment, small gifts are given to these children for gratitude.

The experiment is carried out in a quiet soundproof room, and the subjects have obtained sufficient rest before the experiment. Before the investigation, the subjects were told about the experimental instructions, and the sensors used to measure physiological signals were worn for the subjects. The experiment is conducted after the subjects fully understand the testing process. The selected emotion-induced pictures are presented on the screen in the investigation, and each emotion state includes five images. Each image is shown for 15 s, and the time interval between two pictures is 2 s. The induction time of each emotion is about 1.5 min. Meanwhile, the emotion recognition platform is used to measure the characteristic parameters of 12 physiological signals of the participants in the emotion-induced process. On the one hand, the subjects have 5 min of rest time to calm their emotions after each emotional state induction to avoid the mutual effect of different emotional induction processes. On the other hand, the subjects are induced to generate feelings according to the following order: calmness → happiness → fear, to ensure the practical effect of emotional induction. The whole experiment lasts about 30 min, including oral instruction, registration of measured children, physiological signal acquisition equipment deployment for children, spirited pictures display, data measurement, and the end of recognition after the presentation.

### Experimental Results and Analysis

#### Physiological Signal Characteristic Parameter Sample

**FIGURE 8:**
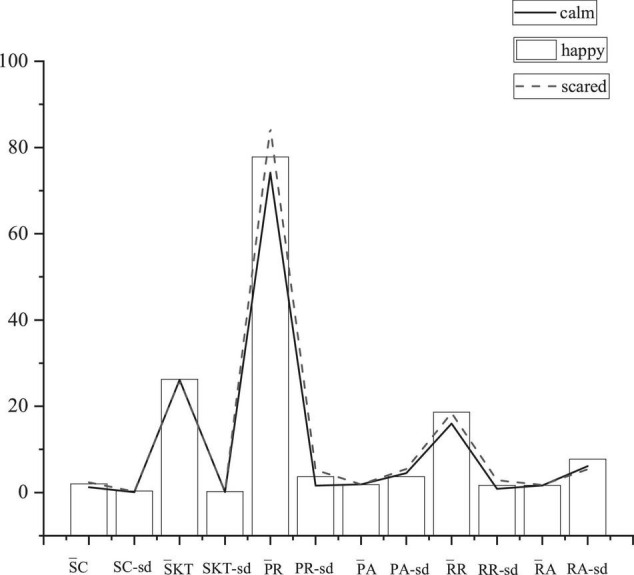
Physiological signal characteristic parameters of a subject.

Through the emotional induction experiment of three kinds of emotions on 20 subjects, 12 physiological signal characteristic parameters of the subjects in three types of emotional state are collected, including $\overset{\_\_\_\_}{PR}$, PR-sd, $\overset{\_\_\_\_}{PA}$, PA-sd, $\overset{\_\_\_\_}{SC}$, SC-sd, $\overset{\_\_\_\_}{RR}$, RR-sd, $\overset{\_\_\_\_}{RA}$, RA-sd, $\overset{\_\_\_\_\_\_\_}{SKT}$, and SKT-sd. There are ultimately 720 valid sample sets of characteristic parameters of physiological signals. Figure 8 reveals 12 physiological signals’ characteristic parameters of a subject in this experiment.

In this experiment, the data samples of 20 subjects, including 10 boys and 10 girls, are divided into two groups. Each group consists of five boys and five girls as the experiment’s training and test samples.

#### Results and Analysis of Emotion Recognition

##### Results of the Emotion Recognition System for the Test Sample Set

**FIGURE 9:**
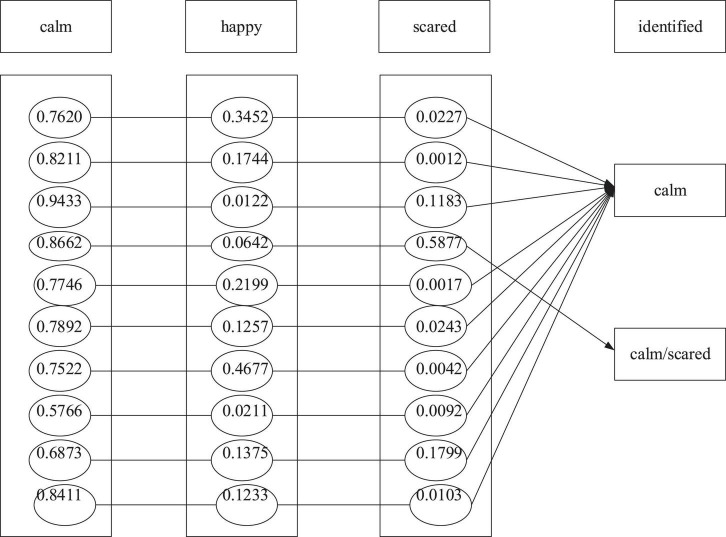
Recognition results under the calm state.

**FIGURE 10:**
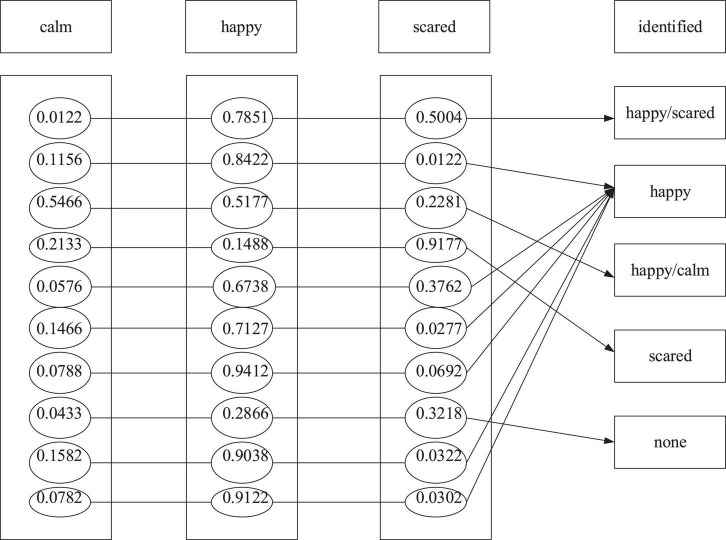
Recognition results under the happy state.

**FIGURE 11:**
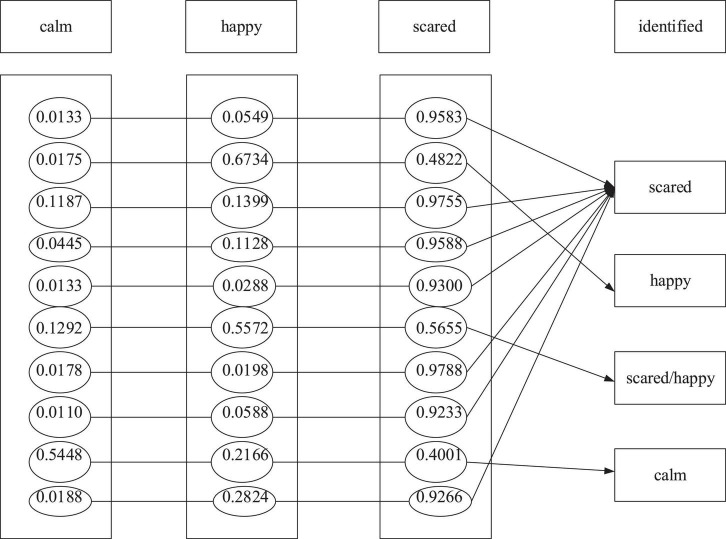
Recognition results under the scared state.

The BPNN model of the emotion recognition system is used to learn the training sample set obtained from the emotion induction experiment to make the BPNN model meet the expected goal. After training the BPNN model, the emotion recognition system recognizes the emotion of the test sample set. The emotional recognition system takes the 12 physiological signal characteristic parameters as the input and the three emotional states of children as output. Denote “1” and “0” as the presence or absence of the emotional state, respectively. Figures 9–11 are the results of children’s recognition under calm, happiness and fear.

In this experiment, the output results are defined as the following: 0.5 is the demarcation point. The output results not less than 0.5 are judged as “1,” indicating that the subject shows the specific emotion. The output result not more than 0.5 is considered “0,” proving that the subject does not offer a particular feeling. In addition, if there are two or more than two output results as “1” simultaneously, it is regarded as a recognition error. Finally, the emotion recognition result is obtained, as shown in the following three figures.

##### Analysis of Children’s Emotion Recognition Ability

From Table 2, regarding the average accuracy of emotion recognition, children aged four to six have particular recognition abilities. Even 4-year-old children can identify emotions to some extent. Intuitively, the emotion recognition ability of children aged 4–6 years old increases with age. The specific situation needs further statistical tests.

**TABLE 2:** Development of children’s emotion recognition (age/gender).

	Age			Gender	
	4 years old	5 years old	6 years old	Boy	Girl
Average recognition rate	0.59 ± 0.19	0.71 ± 0.12	0.81 ± 0.14	0.73 ± 0.21	0.68 ± 0.22

In terms of physiological development, the brain weight of children between 4 and 6 years old increases with age, the synaptic density increases, and the myelination of nerve fibers is completed. Besides, the brain wave level of 6-year-old children accelerates with time (Wei et al., 2019; Wu et al., 2019). The continuous development of the brain also plays a physiological foundation for improving children’s emotion recognition. In terms of speech development, the verbal expression ability of children between 4 and 6 years old increases with age. Since children’s oral understanding is earlier than vocal application and expression, it is difficult to express what they can understand by appropriate words accurately. Furthermore, it is challenging to distinguish emotion recognition ability from verbal in the experimental study. Therefore, the differences in children’s emotion recognition ability are also affected by verbal expressions to some extent (Wu Y. et al., 2020; Yuan and Wu, 2020). As for attention development, 4-year-old children are often unable to focus on the experimental procedure. The performance of attention stability and attention persistence is significantly lower than 5-year-old and 6-year-old children. Besides, teachers or other children are occasionally in the experimental room, and new stimuli often attract children’s attention. Furthermore, compared with children between 5 and 6 years old, 4-year-old children’s attention is easy to disperse. It cannot be maintained in the experimental process, so some children may miss a picture, which affects the recognition results. On the whole, most 6-year-old children can maintain good attention stability and ensure the successful completion of the experiment (Li et al., 2019, 2021).

In summary, children’s emotion recognition ability increases with age. Moreover, their emotion recognition ability generally rapidly develops during 4–6 years old.

##### Analysis of Differences in the Emotion Recognition Ability of Children of Different Ages and Genders

Then, a 3 (ages: four, five, six) were used × 2 (gender: boy and girl) one-way analysis of variance (ANOVA) is used to investigate the age and gender differences in the development of children’s emotional recognition ability. Ages and genders are taken as independent variables, and the average recognition rate of children’s emotion recognition is taken as the dependent variable.

Through Table 3, age has a significant main effect on emotion recognition tasks, with *F* = 16.48 and *p* < 0.05. Meanwhile, the accuracy increases with age. On the contrary, gender does not significantly influence emotion recognition results, with *F* = 3.07 and *p* > 0.05. Furthermore, the interaction between age and gender is insignificant, with *F* = 0.24 and *p* > 0.05. Further analysis indicates that in the emotion recognition task, the emotion recognition ability of children aged 6 is significantly higher than that of children aged 4 (*p* < 0.01) and children aged 5 (*p* < 0.01). Additionally, the emotion recognition ability of children aged 5 is significantly higher than that of children aged 4 (*p* < 0.05).

**TABLE 3:** Test results of the main effect of age on children’s emotion recognition.

Independent variable	SS	df	MS	*F*	Sig.
Age	0.61	3	0.28	16.51	0.000
Gender	0.09	2	0.09	3.11	0.079
Age × gender	0.03	3	0.02	0.19	0.792

The results show that there is no gender difference in recognition of children’s happiness and calm emotion. Still, there is only a marginal significance in the fear emotion, and girls’ recognition of fear is slightly higher than boys.

Through the research of Functional Magnetic Resonance Imaging, there is an interaction effect between the gender difference in recognition of positive and negative emotional content and neural mechanisms. When girls identify positive pictures, they have more activities in the right posterior cingulate gyrus, the left putamen, and the left cerebellum. When they see negative photos, they have more bilateral superior temporal gyrus and cerebellar vermis activities. This demonstrates that girls have more brain activation in emotion recognition and have more advantages. However, children in this experiment do not present significant gender differences. The reason may be that the individual’s brain is in a period of accelerated development in early childhood. Therefore, emotion recognition has not shown gender differences as basic emotional competence. The recognition results of girls are slightly better than those of boys, which may be due to the differences between boys and girls in parenting styles, and girls have better language expression ability, so they can better feel the negative emotions of others in daily life (Zheng et al., 2018; Yuan and Wu, 2020).

##### Analysis of Type Differences in Children’s Emotion Recognition Ability

According to Table 4, children between 4 and 6 years old have a specific ability to identify different types of emotions. The data analysis method of the paired sample *t*-test is used to examine the development order of children’s recognition of different emotions. The results prove that children show apparent advantages in recognizing happiness compared with the other two basic types of feelings. Besides, although the recognition accuracy of fear is lower than that of happiness, it is significantly higher than that of calm. Based on the above data, children’s recognition of happiness is superior to calmness and fear. In summary, children’s recognition ability of three basic emotions from strong to weak is happiness > calm > fear.

**TABLE 4:** Development of children’s emotion recognition (type).

	Happiness	Calmness	Fear	Total points
Average recognition rate	0.91 ± 0.19	0.74 ± 0.16	0.57 ± 0.31	0.73 ± 0.23

To sum up, the development of children’s ability to recognize different types of emotions is unbalanced. According to the development trend analysis, the ability to recognize happiness develops first, and even 4-year-old children can respond to happiness obviously, followed by the ability to acknowledge calmness. The ability to recognize fear develops last. The order of children’s recognition ability of three emotions is as follows: happiness → calm → fear.

##### Research and Analysis of the Theory of Mind in Early Childhood

The subjects are given a card game, followed by two theory of mind tasks in random order. After the game, the mind theory tasks contain the memory test question (Task 1): where did the card start? (Box 1, Box 2, Box 3) and the fact-checking question (Task 2): where is the card now? (Box 1, Box 2, Box 3). On the test questions, the age difference is very significant. Specifically, 5-year-old children are significantly better than 4-year-old children, and 6-year-old children are better than 5-year-old children, indicating that children’s cognitive abilities are getting better as they get older.

To sum up, 4-year-old children are less effective than 5- to 6-year-old children in the theory of mind tasks, which may be related to more complex cognitive processes, such as cognition of different expressions, or the inability to resist the influence of current representation caused by immature representation development of brain inhibition function. The profound reason is that 4-year-old children have limited computing resources, and the growth of children’s computing resources is based on a specific physiological maturity. With certain physiological maturity and the evolution of computing resources, children aged five or six (especially 6-year-old children) can realize that different perception and observation angles will make people have various explanations for the same object or event. Meanwhile, they can understand the relationship between perceptual acquisition of knowledge and knowledge causality, understand the relationship between knowledge, belief, and behavior, and form false opinions about situations.

The emotion recognition experiment is based on ANN, which can obtain more accurate results than traditional recognition experiments with facial images as recognition materials. Scholars Li and Li (2019) proposed a network model that automatically finds faces from pictures or videos and recognizes their expressions. Additionally, they trained the model in the dataset containing face images to automatically detect faces in images and identify their expressions according to faces. Finally, they verified the model’s performance on the tested dataset (Li and Li, 2019). Compared with their research, the present work makes a more detailed analysis of emotion recognition based on a neural network. Moreover, the current work focuses on children. It uses the multi physiological signal emotion recognition system based on ANN to measure children’s multi physiological signals more pertinently. They can show a high recognition rate for their psychological emotions.

#### Discussion on Children’s Emotion Comprehension and the Theory of Mind

The study of emotion has always been a complex and comprehensive field. Emotion recognition of physiological signals provides precious methods and means for studying emotion and has very high application prospect and research value.

The experimental paradigm used here is roughly the same as many classical research models of domestic and foreign scholars. The results show that age is an essential factor affecting the development of children’s theory of mind, and 4 years old is the critical period for developing children’s theory of mind. The experiment confirms that with the growth of age, children complete the task of psychological theory better and better, and their emotional recognition ability is even more robust. This result shows that children’s emotion comprehension is highly consistent with the development of psychological theory, and it is likely that the development of these abilities is interdependent. From the definition of psychological theory and emotion comprehension, it is consistent. Emotion comprehension is the understanding of oneself and others’ emotions. The psychological theory involves individuals’ knowledge of psychology, including visual perception, attention, desire, intention, belief, and related psychological representation and thinking. It is believed that psychological theory is the judgment of internal psychological state and activities, and emotion comprehension can also be understood as cognition. Here, emotion is the material or object of awareness. Individuals judge emotion and behavior caused by emotion combined with the situation and psychological clues. Both emotion comprehension and psychological theory involve individual cognitive ability, and both overlap in a sense in “psychological state” and “cognitive judgment.” Therefore, the correlation between the two is inevitable. This also indicates that the abilities measured by emotion comprehension and psychological theory tasks may promote and depend on each other; that is to say, it is feasible to classify emotion comprehension and psychological theory as two main aspects of social cognition. They jointly represent the development level of individual social understanding.

## Conclusion

Research on emotion is complex and comprehensive. The emotion recognition system based on BPNN reported here provides a precious method and means for people to study emotion, with extensive application prospects and significant research value. The emotion recognition system based on the BPNN model is used to measure the physiological signals of the human body in different emotional states and obtain the required characteristic parameters, to recognize the emotional state of 20 subjects in the experiment. The conclusion is as follows.

(1) The emotion recognition ability of children between 4 and 6 years old increases with age. The order of recognition accuracy of the three emotions is: calmness → happiness → fear. Besides, there is no significant gender difference in facial expression recognition. (2) There are significant age differences in children’s recognition of happiness, calmness, and fear. Children can better identify happiness. Most children over 3 years old can accurately identify happiness, followed by calmness. More than half of children aged 3–4 years old can accurately identify calmness, followed by fear. Children aged 5 years old can identify fear better than calmness. The development trend of recognition of fear, calmness, and happiness is synchronous. Moreover, there is no significant difference in fear emotion recognition of children between 4 and 5 years old, while children aged 6 years old have significant progress.

The BPNN model used in emotion recognition has some limitations, such as the feasibility of network scale and the dependence on training samples. The structure of the BPNN can be designed freely according to users’ needs, and an extensive network scale is often required to solve complex problems. Besides, the physiological signal recognition of emotion is a relatively complex application example. Therefore, when there is an excess of physiological signal parameters and the types of emotional states to be recognized, there are often outstanding requirements for the BPNN model, which will increase the difficulty in the practical application of the BPNN. Correspondingly, future work will explore whether a more suitable ANN model can be applied to emotion recognition by physiological signals. Meanwhile, since preschool is the rapid development stage and key period of emotion recognition, the follow-up work will focus on children’s emotion recognition ability development.

## Data Availability Statement

The raw data supporting the conclusions of this article will be made available by the authors, without undue reservation.

## Ethics Statement

The studies involving human participants were reviewed and approved by the Ethics Committee of China West Normal University. The patients/participants provided their written informed consent to participate in this study. Written informed consent was obtained from the individual(s) for the publication of any potentially identifiable images or data included in this article.

## Author Contributions

All authors listed have made a substantial, direct, and intellectual contribution to the work, and approved it for publication.

## Conflict of Interest

The authors declare that the research was conducted in the absence of any commercial or financial relationships that could be construed as a potential conflict of interest.

## Publisher’s Note

All claims expressed in this article are solely those of the authors and do not necessarily represent those of their affiliated organizations, or those of the publisher, the editors and the reviewers. Any product that may be evaluated in this article, or claim that may be made by its manufacturer, is not guaranteed or endorsed by the publisher.
